# In Vivo Efficacy of SYN023, an Anti-Rabies Monoclonal Antibody Cocktail, in Post-Exposure Prophylaxis Animal Models

**DOI:** 10.3390/tropicalmed5010031

**Published:** 2020-02-21

**Authors:** Tzu-Yuan Chao, Shou-feng Zhang, Li Chen, Eric Tsao, Charles E. Rupprecht

**Affiliations:** 1 Synermore Biologics Co., Ltd., 6F-6, No. 5, Aly. 22, Ln. 513, Ruiguang Rd., Neihu Dist., Taipei 11492, Taiwan; lichen0923@gmail.com (L.C.); etsao@synermore.com (E.T.); 2 Laboratory of Epidemiology and Key Laboratory of Jilin Provincial Zoonosis Control and Prevention, Military Veterinary Research Institute, Academy of Military Medical Sciences, 666 Liuying West Road, Jingyue Economic Development Zone, Changchun, Jilin 130122, China; zhangshoufeng@hotmail.com; 3 LYSSA LLC, Cummings, GA 30040, USA; charles_rupprecht@yahoo.com

**Keywords:** rabies, post-exposure prophylaxis, monoclonal antibody, rabies immune globulin

## Abstract

Rabies immune globulin (RIG) is an indispensable component of rabies post-exposure prophylaxis (PEP) because it provides passive immunity to prevent this otherwise inescapably fatal disease in Category III exposed patients. Even with decades of development, RIG products are still criticized for their high cost, lot-to-lot variation, and potential safety issues. They remain largely unattainable in most developing regions of the world, where demand is highest. In recent years, monoclonal antibodies (MAbs) have become widely accepted as safer and more cost-effective alternatives to RIG products. As an example, SYN023 is a 1:1 cocktail of two humanized anti-rabies MAbs previously shown to display extensive neutralizing capabilities. Here, we further assessed the efficacy of SYN023 in animal models of rabies, and found that SYN023 afforded protection equal to a standard dose of human RIG (HRIG) at 0.03 mg/kg in Syrian hamsters and 0.1 mg/kg in beagles. Potential interference with vaccine-induced immunity was analyzed for the MAbs at these concentrations. While individual MAbs did not interfere with vaccine response, SYN023 at dosages of 0.1 mg/kg and above resulted in reduced neutralizing antibody titers similar to HRIG. Thus, the in vivo characterization of SYN023 supports its utility in human rabies PEP as an efficacious alternative to RIG products.

## 1. Introduction

Rabies is an acute progressive viral encephalomyelitis with the highest case fatality rate of all conventional infectious diseases [1]. This zoonosis is characterized by a progressive infection of the CNS, from the initial site of infection (usually peripheral) to the brain. The disease is caused by viruses belonging to the genus Lyssavirus in the Rhabdoviridae family, the prototype of which is rabies virus (RABV). Exposure to RABV typically occurs via bites or scratches from rabid animals, usually via saliva [2]. Upon virus exposure, timely and proper post-exposure prophylaxis (PEP) can effectively prevent clinical manifestations of rabies. Standard rabies PEP consists of wound care, concurrent administration of rabies immune globulin (RIG) for immediate passive immunity, and rabies vaccine to induce virus-neutralizing antibodies [3]. If administered promptly and appropriately, PEP is highly effective in RABV clearance through local virus neutralization by antibodies or antibody-mediated clearance of virus-infected cells [4]. However, despite a well-defined understanding of the disease pathology and effective prophylaxis, interaction between rabid animals and humans still leads to tens of thousands of human deaths per year worldwide, mostly in Asia and Africa [3]. Despite the production of safe and effective human rabies vaccines, the pressing issue lies in the availability and affordability of RIGs.

The administration of RIG is crucial in conferring passive immunity before the establishment of active immunity against RABV from vaccination. Traditional RIGs used for human rabies PEP are polyclonal immune globulins derived either from the plasma of immunized human donors (HRIG) or from animals, such as horses (equine rabies immunoglobulin, ERIG) [3]. Whilst being highly effective, limited supply in endemic areas, batch-to-batch variability, the cost and the safety of blood-derived products have prompted the search for new products in the prevention of human rabies [5,6]. Several RABV-neutralizing monoclonal antibodies (MAbs) have since been developed by different groups of investigators using hybridoma, phage display, and transgenic mouse technologies [6,7,8,9,10,11]. The recent launch of Rabishield, or SII RMAb, a recombinant human MAb against RABV signifies the advent of a new chapter in rabies PEP, where economical and safe RIG alternatives are used [6,12]. Thus far, the safety and efficacy of Rabishield in rabies PEP have been demonstrated in at least 243 documented cases of Category III RABV exposure in India [12,13]. In a post-market safety study involving 397 subjects, a head-to-head comparison between Rabishield, HRIG, and ERIG revealed no significant differences in their safety profiles [12]. Given the severe burden of rabies in India, the benefits of using Rabishield, a MAb, as a replacement for RIG products may outweigh the risks of limited neutralizing capacity. However, for geographic areas with complex epidemiological scenarios, MAb mixtures targeting at least two distinct antigenic sites will take priority, in line with long-standing World Health Organization (WHO) recommendations [14].

We recently identified and characterized a pair of anti-rabies monoclonal antibodies, CTB011 and CTB012, which were specifically selected for maximal potency and breadth of neutralization against RABVs [15]. As individual MAbs, CTB011 binds RABV glycoprotein at the edge of antigenic site III and CTB012 binds to a novel antigenic site spatially close to site G5; both exhibit nanomolar affinities towards RABV glycoprotein [15]. As a MAb cocktail, the high-affinity complementary binding epitopes ensure broad coverage of representative street RABVs [15]. In a survey of antigenic variation in recent street RABV, the effects of 99 single-point mutations on in vitro neutralization were evaluated for several MAbs and polyclonal antibody preparations [16]. CTB011 and CTB012 were shown to be sensitive to 11 and 4 mutations, respectively, and overlapped minimally on two of the mutations, R333H and R333P, with less than 10-fold reduction in neutralization. Mutations at R333 were highly resistant for polyclonal antibodies as well. The R333P mutant, for example, resulted in 27-fold reduction in neutralization by HRIG, and an 11–220-fold reduction by various pooled antisera. Based on these data, we expect SYN023 to be similar to polyclonal antibodies with respect to the neutralization spectrum against RABV.

While epitope sequence analysis and in vitro neutralization tests provide clues to the breadth of neutralization, the effective concentration required to prevent virus propagation in vivo is more complex. As such, the utility of SYN023 in rabies PEP was further evaluated in Syrian hamsters and beagles against HRIG in the present study. As RIG needs to be administered concurrently with rabies vaccine, the effect of different dosages of SYN023 on vaccine potency was monitored in non-challenged Syrian hamsters. These data are critical for the clinical development of SYN023, which is currently in Phase IIb clinical studies in Category III RABV-exposed patients.

## 2. Materials and Methods 

### 2.1. Challenge Virus Isolate

BD06 (GenBnak: EU549783.1) is a street RABV isolated from a dog in Hebei, China in 2006. As a representative of a Chinese epidemic isolate in viral clade I, it has since been widely utilized both as a vaccine strain and a challenge virus [17].

### 2.2. Biologics

SYN023 was obtained by mixing equal volumes of two anti-RABV MAbs, CTB011 and CTB012, which were produced by Beijing Cotimes Biotech Co., Ltd (China). Rabies vaccine (Rabipur) (lot number 1983 for the Syrian hamster infection study and 1995 for the beagle study) was obtained from Chiron Behring Vaccines Pvt. Ltd., Gujarat, India, and HRIG (lot number 20130306 for the Syrian hamster infection study and 20121103 for the beagle study) was obtained from Guangdong Shuanglin Biological Pharmaceutical Co., Ltd., Zhanjiang, China.

### 2.3. In Vivo Syrian Hamster Challenge Model

Groups of 10 two-month-old female Syrian hamsters were inoculated with street RABV BD06 in the right gastrocnemius muscle, at 100 LD50/50 μL on Day −1. Rabies vaccine was administered to all but the saline control group on Days 0, 3, 7, 14, and 28 in the left gastrocnemius muscle at 50 μL/animal. SYN023 or HRIG was administered into the right gastrocnemius muscle, at the same site as virus inoculation, at 50 μL per animal on Day 0. The test animals were examined two times daily for clinical signs of rabies. Once signs of illness appeared, animals were euthanized via CO_2_ inhalation. The brain tissues were collected from the animals and tested for RABV virus antigens by direct fluorescence antibody (DFA), where tissue samples were stained with a fluorescein-isothiocyanate (FITC)-conjugated anti-rabies antibody [18]. The hamsters were maintained and evaluated for up to 35 days after infection. At the end of the experiment, all survivors were euthanized and the blood was collected by cardiac puncture. Titers of anti-rabies neutralizing antibody (RVNA; IU/mL) in serum samples were determined by the standard rapid fluorescence focus inhibition test (RFFIT) [19].

### 2.4. In Vivo Syrian Hamster Non-Challenge Model

A non-challenge model was performed in Syrian hamsters. All animals except the saline control group were vaccinated on Days 0, 3, 7, 14, 28 at 1% human dose in the left gastrocnemius muscle at 50 μL/animal. The 13 groups of eight hamsters each, received one dose of either HRIG (20 IU/kg), SYN023 (0.003, 0.01, 0.03, 0.1, 0.33, 1 mg/kg), CTB011 (0.05, 0.15, 0.5 mg/kg), or CTB012 (0.05, 0.15, 0.5 mg/kg) on Day 0 in the right gastrocnemius muscle at 0.4 μL/g. Blood samples were collected on Days 1, 3, 7, 14, 28, 42, 60, and 90. The hamsters were maintained and evaluated at up to 90 days. At the end of the experiment, all animals were euthanized and the blood was collected by cardiac puncture. Titers of anti-rabies neutralizing antibody (RVNA; IU/mL) in serum samples were determined by the standard RFFIT [19].

### 2.5. In Vivo Beagle Challenge Model

Three- to six-month-old male and female beagles were inoculated with street RABV BD06 in the masseter muscle at 150,000 LD50 (1mL) on Day −1. They were then randomly assigned to six study groups with two females and two males in each group. Two human doses of rabies vaccine were administered to all but the saline control group at the left and right gastrocnemius muscles, respectively, on Day 0. Two more single doses of rabies vaccine were administered on Days 7 and 21. SYN023 or HRIG was administered into the masseter muscle, at 0.2 mL/kg on Day 0. The test animals were examined two times daily for clinical signs of rabies. Once signs of illness appeared, animals were euthanized and brain tissues were collected for immunofluorescence staining of viral antigens to confirm RABV infection. At the end of the experiment on Day 42, all survivors were euthanized. Euthanasia was achieved through intramuscular injection of anesthetics for deep sedation. 

### 2.6. Ethics Statement

The Syrian hamster infection experiment described in this study was conducted according to the Guidelines on the Human Treatment of Laboratory Animals stipulated by the Ministry of Science and Technology of the People’s Republic of China and approved (license no. 2013–004) by the Animal Welfare Committee of the Military Veterinary Research Institute, Changchun, China. The Syrian hamsters were obtained from the Experimental Animal Center, Changchun Institute of Biological Product (Production license SCXK (Ji) 2012-0001). The use of these animals for this study was approved by the Science and Technology Agency, Jilin Province (IRB).

The non-challenge experiment in Syrian hamsters and the dog experiments were approved by Beijing Cotimes Biotech IRB (protocol # AE-2013-07T and CTB011/CTB012-RD-2014-001). The dogs were supplied by Beijing Rixin Technology Co., Ltd., with production license number of SCXK (Jing) 2011-0007.

All procedures in this study were in accordance with AAALAC standards. Whenever possible, procedures in this study are designed to avoid or minimize discomfort, distress, and pain to animals.

## 3. Results

### 3.1. SYN023 Offers Significant Protection as an In Vivo Model of PEP

SYN023 was previously demonstrated to provide protection in mice challenged with the street RABV isolate BD06 by a mouse neutralization test, suggesting that 12.5 μg of SYN023 (at 0.5 mg/mL) was sufficient to fully neutralize a dose of 100LD50 BD06 [15]. To further characterize the in vivo utility of SYN023 as in PEP for rabies, the MAb cocktail was evaluated in a Syrian hamster model at the dose range of 0.003–1 mg/kg. 

**Figure 1:**
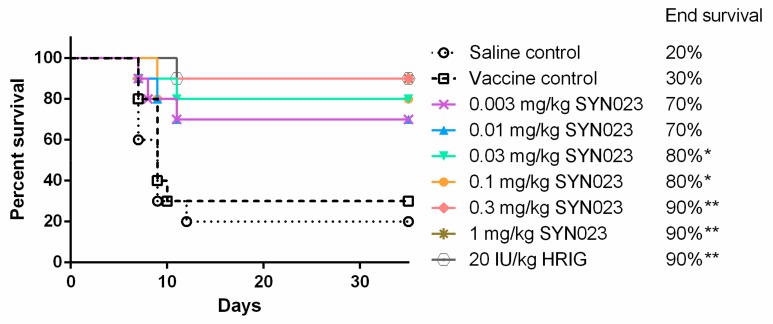
In vivo efficacy of SYN023 in a Syrian hamster post-exposure prophylaxis (PEP) model. Human rabies immune globulin (HRIG) at 20 IU/kg or SYN023 at 0.003, 0.01, 0.03, 0.1, 0.3, and 1 mg/kg were administered in conjunction with rabies vaccine to rabies virus (RABV) (BD06)-infected Syrian hamsters at 24 h post infection. Vaccine was administered on Days 0, 3, 7, 14, and 28. Hamster mortality and morbidity were monitored daily. Vaccine only and untreated groups were included as negative controls. * indicates *p* < 0.05.

In this model, groups of hamsters received full PEP, which included administration of rabies vaccine and either HRIG (20 IU/kg) or SYN023 at different dosages. As shown in Figure 1, SYN023 at 0.03, 0.1, 0.3, and 1 mg/kg resulted in statistically significant survival benefit over saline or vaccine only control groups in a dose-dependent fashion. Standard HRIG administration at 20 IU/kg yielded a survivorship of 90%, which was comparable to a SYN023 dosage of 0.3 mg/kg.

Titers of RVNA were determined for surviving animals by the standard RFFIT upon termination of the study (Table 1). All hamsters treated with the vaccine were positive for RVNA. Groups with higher survival rates exhibited slightly lower mean RVNA levels. SYN023 at 0.3 mg/kg was comparable to HRIG at 20 IU/kg.

### 3.2. Effects of CTB011, CTB012, and SYN023 on Serum RVNA Levels in Syrian Hamsters

**Figure 2:**
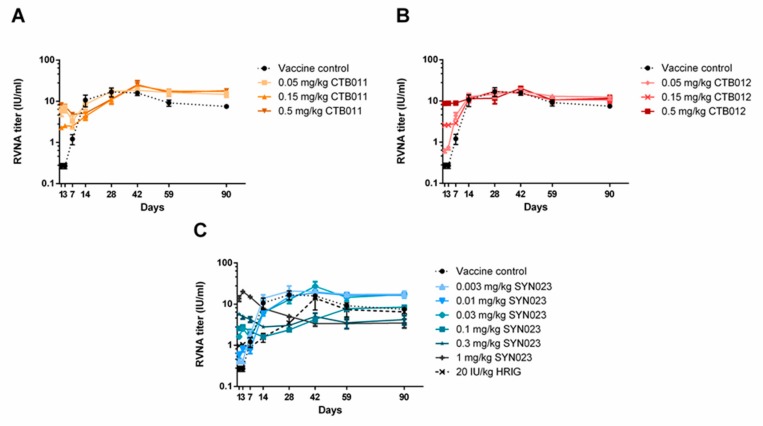
Changes of serum anti-rabies antibody titers in non-challenged Syrian hamsters. (**A**) CTB011 (0.05, 0.15, 0.5 mg/kg), (**B**) CTB012 (0.05, 0.15, 0.5 mg/kg), (**C**) SYN023 (0.003, 0.01, 0.03, 0.1, 0.33, 1 mg/kg), or HRIG (20 IU/kg) were administered to the opposite site of vaccination on Day 0. Serum RVNA levels were monitored by performing a rapid fluorescence focus inhibition test (RFFIT) on blood samples.

Potential interference should be thoroughly characterized for consideration of an optimal dosage, so, we examined the effect of SYN023 as well as its component MAbs, CTB011 and CTB012 in Syrian hamsters in the absence of a virus challenge. As shown in Figure 2A,B, administration of MAb CTB011 or CTB012 alone had minimal interference effects as their RVNA profiles overlapped significantly with that of the vaccine control group. SYN023 at 0.03, 0.01, and 0.003 mg/kg also had no observable interference with antibody formation (Figure 2C). In contrast, lower mean RVNA titers were observed for higher dosage groups of SYN023, starting at 0.1 mg/kg, particularly on Days 14 and 28. The MAb cocktail had greater interference than individual MAbs at similar concentrations. The lower dosage groups 0.03, 0.01, and 0.003 mg/kg were able to maintain higher RVNA titers than the vaccine control group from Day 28 to Day 90. The 20 IU/mL HRIG group also had lower mean RVNA titers on Days 14 and 28 in comparison to the vaccine group.

### 3.3. SYN023 Protects Beagles from a Lethal Challenge of BD06 Street RABV

**Figure 3:**
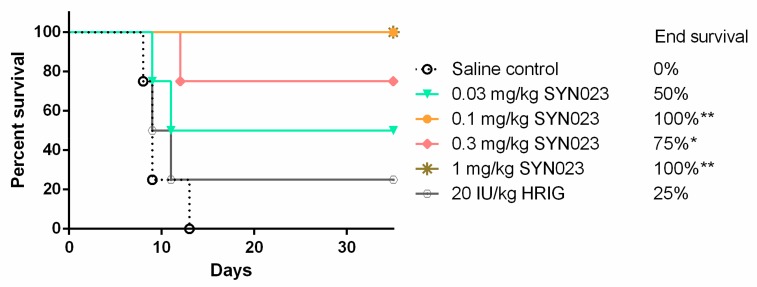
In vivo efficacy of SYN023 in a beagles PEP model. HRIG at 20 IU/kg or SYN023 at 0.03, 0.1, 0.3, and 1 mg/kg were administered in conjunction with rabies vaccine to RABV (BD06)-infected Syrian hamsters at 24 h post infection. Two doses of vaccine were administered on Day 0, followed by one dose each on Days 7 and 21. Animal mortality and morbidity were monitored daily. Saline group was included as negative control. * indicates *p* < 0.05.

We tested SYN023 in a different model of PEP in dogs. The dogs were infected with the Chinese street RABV BD06, and received either SYN023 (0.03, 0.1, 0.3 and 1 mg/kg) or HRIG (20 IU/kg) and vaccine as PEP. As shown in Figure 3, no survival of controls and low survival of the HRIG group indicate that the dose was in line with a severe viral challenge. However, despite the high virus inoculum, SYN023 at 0.1 mg/kg and 1 mg/kg offered complete protection against BD06. All of the SYN023 treatment groups provided better protection to the animals than 20 IU/kg HRIG in this study. 

## 4. Discussion

The use of passive immunization in rabies PEP dates back to the late 19th century, where supplementation of vaccine with RABV neutralizing serum was first described by Babes and Lepp [20]. The pronounced efficacy demonstrated in that work subsequently led to the establishment and wide recognition of combining active vaccination with RABV antiserum. By the 1950s, and after a trial in Iran, the use of antiserum with vaccine had become the standard of care for RABV Category III exposures [6]. In 1974, the first commercial RIG preparation was launched, and has since been co-evolving with the biotech industry [1]. Today, over 11 ERIG and 12 human plasma-derived RIG are available. Regardless, each year, of the 20 million RABV-exposed patients worldwide in need of PEP, only less than 2% of Category III exposed patients receive RIG [21]. Reasons for the low adoption of RIG include cost, safety, and supply. As strongly advocated by the WHO, anti-rabies MAbs have been proposed as a practical alternative to RIG to fulfil these unmet medical needs, particularly in lesser developed countries [21]. 

CTB011 and CTB012 are a pair of humanized IgGs that bind to non-overlapping antigenic sites on the RABV glycoprotein [15]. The MAb cocktail, SYN023, which consists of an equal molar mixture of CTB011 and CTB012 was previously found to exhibit a broad spectrum of neutralization against panels of street RABV strains in neutralization assays (i.e., rapid fluorescence focus inhibition test, RFFIT) and in mouse neutralization tests [15]. In an experimental PEP model in Syrian hamsters, SYN023 at 0.03 mg/kg was shown to have equivalent protection to 20 IU/kg HRIG against a *Tadarida brasiliensis* bat RABV [15]. In the present study against the BD06 RABV, we found that SYN023 at 0.03–1 mg/kg provided statistically significant benefits to survivorship (80–90%), similar to the 90% survivorship achieved with a standard HRIG dose (Figure 1). BD06 is a well-characterized RABV in China and is considered to be highly virulent [17,22]. Given this, a higher concentration of SYN023 might be required for the neutralization of a more virulent RABV. We then further tested SYN023 in a PEP model in dogs, again infected with BD06 RABV. As shown in Figure 3, SYN023 at 0.03, 0.1, 0.3, and 1 mg/kg in combination with vaccine resulted in 50%, 100%, 75%, and 100% survival, respectively. Altogether, accumulated data obtained from various PEP models suggest that SYN023 at 0.03 mg/kg in Syrian hamsters and 0.1 mg/kg in beagles provides similar protection to HRIG against multiple RABV. Coincidentally, a dose of 0.03 mg/kg in Syrian hamsters is in the same order of magnitude as the HRIG equivalent dose determined for two other anti-rabies MAb cocktails, CL184 (0.012 mg/kg) and RVC20/RVC58 (0.045 mg/kg) [23,24]. As the efficacy of MAbs are highly contingent on specific mutations associated with each RABV, a side-by-side comparison of different MAb pairs against the same panel of viruses would yield more precise results.

Besides efficacy, the degree of vaccine interference is another important consideration in dosage selection. As illustrated in studies investigating the impact of different HRIG dosages on neutralizing antibody response, 40 IU/kg was consistently found to correlate with significantly reduced RVNA titers [20,25,26]. By contrast, mixed results were obtained for the WHO recommended dosage of 20 IU/kg—interference was evident in some studies [27,28] but minimal in others [26,29,30]. However, irrespective of the duration and intensity of vaccine interference introduced by concomitant RIG administration, all reported RVNA levels were still maintained at above 0.5 IU/ml between Day 14 and Day 90, which is considered indicative of an adequate immune response to vaccination [21]. 

In the present study, we thoroughly examined potential vaccine interference activities of SYN023 in both virus challenge and non-challenge models. In the BD06 RABV infection protection experiment in Syrian hamsters, terminal RVNA titers were analyzed and were found to be directly related to dose levels; no vaccine interference was found for lower dosages up to 0.1 mg/kg. Some interference was observed for two higher dosages at 0.3 and 1 mg/kg, which was highly comparable to HRIG at 20 IU/mL (Table 1). In the non-infection model, RVNA titers were monitored for CTB011, CTB012, and SYN023 for 90 days (Figure 2). The RVNA profile for CTB012 was essentially indistinguishable from that of the vaccine control group from Day 14 to Day 90 at all dosage levels, 0.05, 0.15, and 0.5 mg/kg. Alternatively, the RVNA profiles for CTB011 groups were slightly lower on Days 14 and 28 and leveled off at higher levels in comparison to the vaccine control group. Given that the two MAbs have nearly identical binding affinities towards RABV glycoprotein, location of the epitopes seems to have led to different RVNA profiles. Importantly, neither of the MAbs exhibited interference to a significant extent at up to 0.5 mg/kg. Nonetheless, when combined together, significantly reduced RVNA titers were observed during Day 14 to 42 for SYN023 at 0.1, 0.3, and 1 mg/kg. Some interference was noted for HRIG at 20 IU/kg during Day 14 to 28. The low RVNA titers observed prior to Day 7 suggest that the MAb content of 20 IU/kg HRIG was comparable to 0.01–0.03 mg/kg SYN023. However, the interference on Day 14 was comparable to 0.1–0.3 mg/kg SYN023, which was likely a result of its polyclonal nature. Collectively, these data suggest that dosage levels, and the number and location of the binding epitope are all primary determinants of vaccine interference. 

The approval of SII RMab or Rabishield marked an important milestone in the transition from blood-derived human immune globulins to stable cell line-produced MAbs. With several MAb cocktails under development, the next challenge will be to match or exceed the neutralization spectrum of polyclonal RIG products with two MAbs. Both the multispecies wildlife reservoirs and high mutation rates of RABV will keep driving antigenic changes within the lyssavirus genus [16]. Thus, the optimal dosage of SYN023 will be a balance between extending ideal neutralizing capacities to multiple lyssaviruses while minimizing vaccine interference concomitantly during human PEP. In addition, although all domestic species at risk should receive pre-exposure vaccination, based upon the considerable protection demonstrated in a highly relevant animal model, the use of MAbs for prophylaxis in veterinary medicine for management of the naïve individual should be further explored within a One Health context [31].

## Figures and Tables

**Table 1:** Serum anti-rabies neutralizing antibody (RVNA) titer in surviving animals.

Group No.	Treatment	Survival	Serum RVNA Levels Determined by RFFIT (IU/mL)									
			#1	#2	#3	#4	#5	#6	#7	#8	#9	Mean ± SD ^1^
**1**	Saline	2	0	0	−	−	−	−	−	−	−	0
**2**	Vaccine	3	14	4.0	7.8	−	−	−	−	−	−	8.7 ± 5.2
**3**	0.003 mg/kg SYN023	7	8.3	12	12	5.7	5.6	19	2.9	−	−	9.4 ± 5.5
**4**	0.01 mg/kg SYN023	7	9.2	9.2	11	7.9	3.1	11	9.7	−	−	8.7 ± 2.7
**5**	0.03 mg/kg SYN023	8	8.7	5.5	10	8.8	12	6.7	11	9.6	−	9.1 ± 2.2
**6**	0.1 mg/kg SYN023	8	9.7	5.2	9.0	8.3	4.4	11	15	7.9	−	8.9 ± 3.4
**7**	0.3 mg/kg SYN023	9	4.7	11	4.2	7.5	9.4	19	5.3	2.8	3.8	7.5 ± 3.3
**8**	1 mg/kg SYN023	9	3.7	9.4	5.0	7.8	10.	14	5.4	5.2	4.3	7.2 ± 3.3
**9**	20 IU/mL HRIG	9	13	9.1	3.8	13	6.0	3.5	7.8	7.7	2.8	7.4 ± 3.9

^1^ No statistical significance between vaccine control and treatment groups RVNA mean titers by one-way ANOVA.

## References

[1] Rupprecht C.E., Hanlon C.A., Hemachudha T. (2002). Rabies re-examined. Lancet Infect. Dis..

[2] Rabies. https://www.who.int/news-room/fact-sheets/detail/rabies.

[3] World Health Organization (2018). WHO Expert Consultation on Rabies, Third Report. https://apps.who.int/iris/bitstream/10665/85346/1/9789241209823_eng.pdf.

[4] Dietzschold B., Kao M., Zheng Y.M., Chen Z.Y., Maul G., Fu Z.F., Rupprecht C.E., Koprowski H. (1992). Delineation of putative mechanisms involved in antibody-mediated clearance of rabies virus from the central nervous system. Proc. Natl. Acad. Sci. USA.

[5] Why a Simple, Lifesaving Rabies Shot Can Cost $10,000 in America. https://www.vox.com/policy-and-politics/2018/2/7/16851134/rabies-treament-expensive-emergency-room.

[6] Sparrow E., Torvaldsen S., Newall A.T., Wood J.G., Sheikh M., Kieny M.P., Abela-Ridder B. (2019). Recent advances in the development of monoclonal antibodies for rabies post exposure prophylaxis: A review of the current status of the clinical development pipeline. Vaccine.

[7] Müller T., Dietzschold B., Ertl H., Fooks A.R., Freuling C., Fehlner-Gardiner C., Kliemt J., Meslin F.X., Franka R., Rupprecht C.E. (2009). Development of a mouse monoclonal antibody cocktail for post-exposure rabies prophylaxis in humans. PLoS Negl. Trop. Dis..

[8] Nagarajan T., Rupprecht C.E., Dessain S.K., Rangarajan P.N., Thiagarajan D., Srinivasan V.A. (2008). Human monoclonal antibody and vaccine approaches to prevent human rabies. Curr. Top. Microbiol. Immunol..

[9] Bakker A.B., Marissen W.E., Kramer R.A., Rice A.B., Weldon W.C., Niezgoda M., Hanlon C.A., Thijsse S., Backus H.H., De Kruif J. (2005). Novel human monoclonal antibody combination effectively neutralizing natural rabies virus variants and individual in vitro escape mutants. J. Virol..

[10] Sloan S.E., Hanlon C., Weldon W., Niezgoda M., Blanton J., Self J., Rowley K.J., Mandell R.B., Babcock G.J., Thomas W.D. (2007). Identification and characterization of a human monoclonal antibody that potently neutralizes a broad panel of rabies virus isolates. Vaccine.

[11] Nagarajan T., Marissen W., Rupprecht C.E. (2014). Monoclonal antibodies for the prevention of rabies: Theory and clinical practice. Antib. Technol. J..

[12] Shivalingaiah A.H., Shankaraiah R.H., Hanumanthaiah A.N.D. (2018). Safety of new indigenous human Rabies Monoclonal Antibody (RMAb) for post exposure prophylaxis. Indian J. Community Health.

[13] Gogtay N.J., Munshi R., Narayana D.H.A., Mahendra B.J., Kshirsagar V., Gunale B., Moore S., Cheslock P., Thaker S., Deshpande S. (2018). Comparison of a novel human rabies monoclonal antibody to human rabies immunoglobulin for postexposure prophylaxis: A phase 2/3, randomized, single-blind, noninferiority, controlled study. Clin. Infect. Dis..

[14] Ilina E.N., Larina M.V., Aliev T.K., Dolgikh D.A., Kirpichnikov M.P. (2018). Recombinant monoclonal antibodies for rabies post-exposure prophylaxis. Biochemistry.

[15] Chao T.Y., Ren S., Shen E., Moore S., Zhang S.F., Chen L., Rupprecht C.E., Tsao E. (2017). SYN023, a novel humanized monoclonal antibody cocktail, for post-exposure prophylaxis of rabies. PLoS Negl. Trop. Dis..

[16] Wang W., Ma J., Nie J., Li J., Cao S., Wang L., Yu C., Huang W., Li Y., Yu Y. (2019). Antigenic variations of recent street rabies virus. Emerg. Microbes Infect..

[17] Liu Y., Zhang S.F., Pan T.L., Zhang F., Wang Y., Lian H., Zhang J.X., Hu R.L. (2014). Characteristics of rabies street virus strain BD06 for challenge test in dogs. Chin. J. Biol..

[18] Dean D.J., Abelseth M.K., Atanasiu P., Meslin F.X., Kaplan M.M., Koprowski H. (1996). The Fluorescent Antibody Test. Laboratory Techniques in Rabies.

[19] Smith J.S., Yager P.A., Baer M., Meslin F.X., Kaplan M.M., Koprowski H. (1996). A rapid fluorescent focus inhibition test (RFFIT) for determining rabies virus-neutralizing antibody. Laboratory Techniques in Rabies.

[20] Cabasso V.J., Loofbourow J.C., Roby R.E., Anuskiewicz W. (1971). Rabies immune globulin of human origin: Preparation and dosage determination in non-exposed volunteer subjects. Bull. World Health Organ..

[21] WHO (2018). Rabies Vaccines: WHO Position Paper-April 2018. Wkly. Epidemiol. Rec..

[22] Wang X., Zhang S., Sun C., Yuan Z.G., Wu X., Wang D., Ding Z., Hu R. (2011). Proteomic profiles of mouse neuro N2a cells infected with variant virulence of rabies viruses. J. Microbiol. Biotechnol..

[23] Franka R., Carson W.C., Ellison J.A., Taylor S.T., Smith T.G., Kuzmina N.A., Kuzmin I.V., Marissen W.E., Rupprecht C.E. (2017). In vivo efficacy of a cocktail of human monoclonal antibodies (CL184) against diverse North American bat rabies virus variants. Trop. Med. Infect. Dis..

[24] De Benedictis P., Minola A., Rota Nodari E., Aiello R., Zecchin B., Salomoni A., Foglierini M., Agatic G., Vanzetta F., Lavenir R. (2016). Development of broad-spectrum human monoclonal antibodies for rabies post-exposure prophylaxis. EMBO Mol. Med..

[25] Warrell M.J., Warrell D.A., Suntharasamai P., Viravan C., Sinhaseni A., Udomsakdi D., Phanfung R., Xueref C., Vincent-Falquet J.C., Nicholson K.G. (1983). An economical regimen of human diploid cell strain anti-rabies vaccine for post-exposure prophylaxis. Lancet.

[26] Helmick C.G., Johnstone C., Sumner J., Winkler W.G., Fager S. (1982). A clinical study of Merieux human rabies immune globulin. J. Biol. Stand..

[27] Vodopija I., Sureau P., Smerdel S., Lafon M., Baklaić Z., Ljubicić M., Svjetlicić M. (1988). Interaction of rabies vaccine with human rabies immunoglobulin and reliability of a 2-1-1 schedule application for postexposure treatment. Vaccine.

[28] Lang J., Simanjuntak G.H., Soerjosembodo S., Koesharyono C. (1998). Suppressant effect of human or equine rabies immunoglobulins on the immunogenicity of post-exposure rabies vaccination under the 2-1-1 regimen: A field trial in Indonesia. Bull. World Health Organ..

[29] Suntharasamai P., Chaiprasithikul P., Wasi C., Supanaranond W., Auewarakul P., Chanthavanich P., Supapochana A., Areeraksa S., Chittamas S., Jittapalapongsa S. (1994). A simplified and economical intradermal regimen of purified chick embryo cell rabies vaccine for postexposure prophylaxis. Vaccine.

[30] Hanna K., Cruz M.C., Mondou E., Corsi E., Vandeberg P. (2018). Safety and neutralizing rabies antibody in healthy subjects given a single dose of rabies immune globulin caprylate/chromatography purified. Clin. Pharmacol..

[31] Wilson P.J., Oertli E.H., Hunt P.R., Sidwa T.J. (2010). Evaluation of a postexposure rabies prophylaxis protocol for domestic animals in Texas: 2000–2009. J. Am. Vet. Med. Assoc..

